# Functional characterization and reconstitution of ABA signaling components using transient gene expression in rice protoplasts

**DOI:** 10.3389/fpls.2015.00614

**Published:** 2015-08-05

**Authors:** Namhyo Kim, Seok-Jun Moon, Myung K. Min, Eun-Hye Choi, Jin-Ae Kim, Eun Y. Koh, Insun Yoon, Myung-Ok Byun, Sang-Dong Yoo, Beom-Gi Kim

**Affiliations:** ^1^Molecular Breeding Division, National Academy of Agricultural Science, Rural Development AdministrationJeonju, South Korea; ^2^Department of Life Sciences, Korea UniversitySeoul, South Korea

**Keywords:** ABA, rice protoplast, reconstitution of signaling, transient expression, dual luciferase assay

## Abstract

The core components of ABA-dependent gene expression signaling have been identified in *Arabidopsis* and rice. This signaling pathway consists of four major components; group A OsbZIPs, SAPKs, subclass A OsPP2Cs and OsPYL/RCARs in rice. These might be able to make thousands of combinations through interaction networks resulting in diverse signaling responses. We tried to characterize those gene functions using transient gene expression for rice protoplasts (TGERP) because it is instantaneous and convenient system. Firstly, in order to monitor the ABA signaling output, we developed reporter system named pRab16A-fLUC which consists of *Rab16A* promoter of rice and luciferase gene. It responses more rapidly and sensitively to ABA than pABRC3-fLUC that consists of ABRC3 of *HVA1* promoter in TGERP. We screened the reporter responses for over-expression of each signaling components from group A OsbZIPs to OsPYL/RCARs with or without ABA in TGERP. OsbZIP46 induced reporter most strongly among OsbZIPs tested in the presence of ABA. SAPKs could activate the OsbZIP46 even in the ABA independence. Subclass A OsPP2C6 and -8 almost completely inhibited the OsbZIP46 activity in the different degree through the SAPK9. Lastly, OsPYL/RCAR2 and -5 rescued the OsbZIP46 activity in the presence of SAPK9 and OsPP2C6 dependent on ABA concentration and expression level. By using TGERP, we could characterize successfully the effects of ABA dependent gene expression signaling components in rice. In conclusion, TGERP represents very useful technology to study systemic functional genomics in rice or other monocots.

## Introduction

Living organisms use receptors to recognize factors in the extracellular environment such as light, water, and pathogens. Receptors trigger biochemical events that transduce the signal to the inside of cell. In response to such signals, metabolism and gene expression within cells are altered. Plants are sessile organisms that cannot move away from adverse environments toward favorable environments. Thus, plants are exposed to much more diverse environmental conditions compared to animals and have to respond and adapt to adverse environments to survive. Therefore, it might be supposed that plants have more complex signal transduction systems than animals. Indeed, several unique molecular systems to transduce signals are present in plants (Trewavas, 2002).

One of the biggest challenges looming for agricultural research and policy is how to feed the estimated nine billion people on the planet in 30 years, especially in the phase of global warming (Gregory and George, 2011; Springer and Duchin, 2014). Accordingly, one of the major concerns of crop research scientists is to increase crop productivity in adverse environments. For this, it is necessary to understand the molecular mechanisms underlying signal transduction related to abiotic stress and crop productivity. Thus, methodologies to monitor signal sensing, transduction and output are required. Among several types of signal outputs, gene expression and protein synthesis alteration are the most suitable outputs which monitor the effects of signaling. Reporter systems for gene expression consist of promoters of genes regulated by target signals and reporter genes such as *chloramphenicol acetyltransferase* (CAT), *beta-glucuronidase* (β-GUS), *beta-galactosidase* (β-GAL), *luciferase* (LUC), and *green fluorescent protein* (GFP), (Peach and Velten, 1992; Cormack et al., 1998; Hammerling et al., 1998; Blazquez, 2007). Recently LUC has been most often used as a transcription monitoring reporter in plants and animals because LUC has a short turnover time and high sensitivity (Yoo et al., 2007).

It takes much time and effort to develop whole-plant transcriptional assay systems. Thus, transient gene expression systems are often used to study signaling in plants. For these purposes, agro-infiltration and transient gene expression systems in protoplasts are most commonly applied in dicot plants (Sainsbury and Lomonosoff, 2014). Transient gene expression in protoplasts also has been performed in monocots such as rice and maize. However, the efficiency of protoplast isolation and transformation was low and large amounts of protoplasts were required because of low-sensitivity of reporter systems. Recently efficient protoplast isolation methods were reported and successfully used for cell biology studies such as subcellular localization and cellular interaction analyses, including BiFC, in rice (Chen et al., 2006; Zhang et al., 2011). These advances suggest that rice protoplasts might be useful to monitor gene expression and identify gene functions in signaling pathways (Sheen, 2001).

ABA plays important roles in abiotic stress tolerance of plants. Recently ABA signaling components that regulate ABA-dependent gene expression were identified from receptors to transcription factors in *Arabidopsis* and rice (Park et al., 2009; Umezawa et al., 2009; Kim et al., 2012; Soon et al., 2012). When the ABA concentration in the cell goes up, ABA receptors PYL/RCAR bind ABA and interact with subclass A PP2Cs, which normally suppress SnRK2. As a result, SnRK2 activates bZIP transcription factors by phosphorylation and ABA-dependent gene expression is activated in *Arabidopsis* (Xiang et al., 2008; Kulik et al., 2011). In rice, the orthologs of these signaling components have been identified by bioinformatics (Kim et al., 2012; He et al., 2014). Rice contains 10 OsPYL/RCARs, 9 subclass A PP2Cs, 10 SAPK (Stress/ABA-activated protein kinases) and 10 group A bZIP transcription factors (Kim et al., 2012). The ABA signaling pathway of *Arabidopsis* was successfully reconstituted via transient expression in *Arabidopsis* mesophyll cell protoplasts (Fujii et al., 2009). However, in rice the functions of few ABA signaling components genes have been confirmed and the signaling pathway has not been reconstituted yet.

In this study, we developed the system of a transient gene expression for rice protoplasts (TGERP), reconstituted the ABA signaling components using TGERP and characterized the effects of components in ABA signaling through monitoring gene expression based on the LUC reporter. This system is suitable for high-throughput analysis because it generates data rapidly, quantitatively and inexpensively. Thus, it represents valuable technology for functional genomics approaches in the post-genomic era of rice.

## Materials and Methods

### Plant Material and Growth Conditions

Rice (*Oryza sativa* cv. Dongjin) dehulled seeds were sterilized with 70% ethanol for 1 min followed by 50% sodium hypochlorite of for 40 min and thoroughly washed 5–6 times with sterile distilled water. To isolate protoplasts, these seeds were grown on 1/2 Murashige and Skoog (MS) medium and initially kept under dark conditions for 8–10 days to induce long stems before being placed under long-day conditions (16 h light and 8 h dark) for 1–2 days at 28°C.

### Rice Protoplast Isolation and Transfection

Rice protoplast isolation methods were reported by several research groups (Chen et al., 2006; Zhang et al., 2011; Kim et al., 2012). We modified those methods and optimized them as follows. A bundle of rice seedlings (about 36 seedlings) were chopped into 0.5–1 mm strips using a surgical blade. Chopped seedlings were quickly transferred to freshly prepared enzyme solution (1.5% cellulose R-10, 0.75% macerozyme R-10, 0.6 M mannitol, 10 mM MES at pH 5.7, 0.1% BSA, 3.4 mM CaCl_2_, 5 mM β-mercaptoethanol, and 50 μL mL^-1^ ampicillin) and soaked for 3–4 h in the dark with gentle shaking (50 rpm). After enzymatic digestion, the enzyme solution containing protoplasts was diluted with three volumes of W5 solution (0.1% glucose, 0.9% NaCl, 2 mM MES, 0.08% KCl, and 125 mM CaCl_2_ at pH 5.65) before filtration to remove undigested stem tissues. Diluted protoplasts were filtered through 145-μm mesh into 50-mL conical tubes. The protoplasts were collected by centrifugation at 100 *g* for 10 min at 28°C. After washing, collected protoplasts were re-suspended in 4 mL W5 solution, and then re-suspended protoplasts were floated on 5 mL 22% sucrose to separate burst protoplasts. After centrifugation, intact protoplasts were collected from the green layer between the sucrose and the W5. After intact protoplasts were washed one more time with W5 solution, the protoplasts were re-suspended in MaMg solution (600 mM mannitol, 15 mM MgCl_2_, and 5 mM MES at pH 5.65). For transfections, 300 μL protoplasts were mixed with plasmid constructs and 330 μL PEG solution [400 mM mannitol, 100 mM Ca(NO_3_)_2_, and 40% PEG-6000]. The mixture was incubated for 30 min at 28°C. After incubation, W5 solution was added stepwise to dilute the PEG solution. Protoplasts were collected by centrifugation at 100 g for 10 min at 28°C. Supernatants were removed and the protoplasts were re-suspended in W5 solution and incubated.

### Subcellular Localization and Bimolecular Fluorescence Complementation Assay

For subcellular localization analysis, the sequences encoding OsbZIPs, SAPKs, OsPP2Cs, and OsPYL/RCARs were amplified by PCR with specific primers, and PCR products were inserted into the pENTR/D/TOPO vector (Invitrogen, USA). The products were recombined into pMDC43 or pMDC83 vectors using LR Clonase (Invitrogen, USA). The TOPO cloning and LR reactions were carried out according to the manufacturer’s instructions (Invitrogen, USA). The plasmids (10 μg) were introduced into rice seedling protoplasts by PEG-mediated transfection. GFP fluorescence was observed and images were captured with an Axioplan fluorescence microscope (AxioImager M1, Carl Zeiss, Jena, Germany).

For BiFC assays, coding sequences for SAPK9, OsPYL/RCAR2, and OsPYL/RCAR5 were cloned into the pVYCE vector resulting in fusion with the C-terminus of the yellow fluorescent protein (YFP). Coding sequences for OsPP2C6 and OsPP2C8 were cloned into pVYNE vector, resulting in fusion with the N-terminus of the YFP sequence (Waadt et al., 2008). Rice protoplasts were transfected with plasmid combinations (15 μg each) of fluorescent protein fragments by PEG-mediated transfection. Reconstituted YFP fluorescence was observed and images were captured with an Axioplan fluorescence microscope (AxioImager M1, Carl Zeiss, Jena, Germany) at 16–24 h incubation. In both experiments, 1–5 × 10^6^ cells mL^-1^ protoplasts were used.

### Construction of Reporter Vector for Dual-Luciferase Assays

To construct an ABA-responsive reporter plasmid vector consisting of the *Rab16A* promoter fused with firefly luciferase (fLUC), we amplified the *Rab16A* (Loc_Os11g26790) promoter region including 91 bp of 5′UTR by PCR from *Oryza sativa* cv. Dongjin genomic DNA with specific primers (Rab16A–F, 5′-CTGAGAGAGGATGACCCT TGTCACC-3′; Rab16A-R, 5′-TTTGGCGTCTTCCATCCTGCTTAAGCTAAAGCTGA-3′), and the fLUC gene including the Nos terminator region was amplified by PCR from the pABRC3-fLUC reporter plasmid with specific primers (fLUC-F, 5′-TTTAGC TTAAG CA GGATGGAAGACGCCAAAAACATAAAGAAAGGCCCGC-3′; NosT-R, 5′-GATCTAGT AACATAGATGACACCGCGCGCG-3′). These two PCR products were re-amplified using Rab16A-F and NosT-R primers. The final PCR products were cloned into pCRTM8/GW/TOPO vector (Invitrogen, USA), and the resulting reporter vector was named as pRab16A-fLUC (**Supplementary Figure S1**).

### Dual-Luciferase Assays

For dual-luciferase assays, coding sequences for OsbZIPs and OsPP2Cs were cloned into the transient expression vector pGEM-UbiHA, which contains the maize *ubiquitin* promoter and sequence encoding a 3XHA tag. Coding sequences for SAPKs and OsPYL/RCARs were cloned into the transient expression vector pGEM-UbiFlag resulting in fusion with the flag tag. The resulting effector plasmids were used for rice protoplast transfection. After transfection, transfected protoplast cells were divided into two samples and incubated in W5 solution with or without ABA. After incubation, the protoplasts were harvested, frozen in liquid nitrogen and stored at –80°C. The frozen protoplasts were re-suspended in 100 μL Passive lysis buffer (Promega, USA). Reporter activities were measured in 10 μL lysate using a dual luciferase assay system according to the manufacturer’s instructions (Promega, USA). The pRab16A-fLUC and pABRC3-fLUC constructs were used as ABA-responsive reporters (8 μg plasmid per transfection). pAtUBQ-rLUC (*Renilla* luciferase) was added to each sample as an internal control (1 μg per transfection; **Supplementary Figure S1**). OsbZIP-HA, SAPK-Flag, OsPP2C-HA, and OsPYL/RCAR-Flag effector plasmids were used at 10 μg per transfection. The relative luciferase activity [fLUC/(*Renilla* luciferase/*Renilla* luciferase average)] was calculated to normalize values after each assay.

## Results

### Rab16A Promoter Fused to Luciferase is Suitable as a Gene Expression Reporter for ABA Signaling in TGERP

**FIGURE 1:**
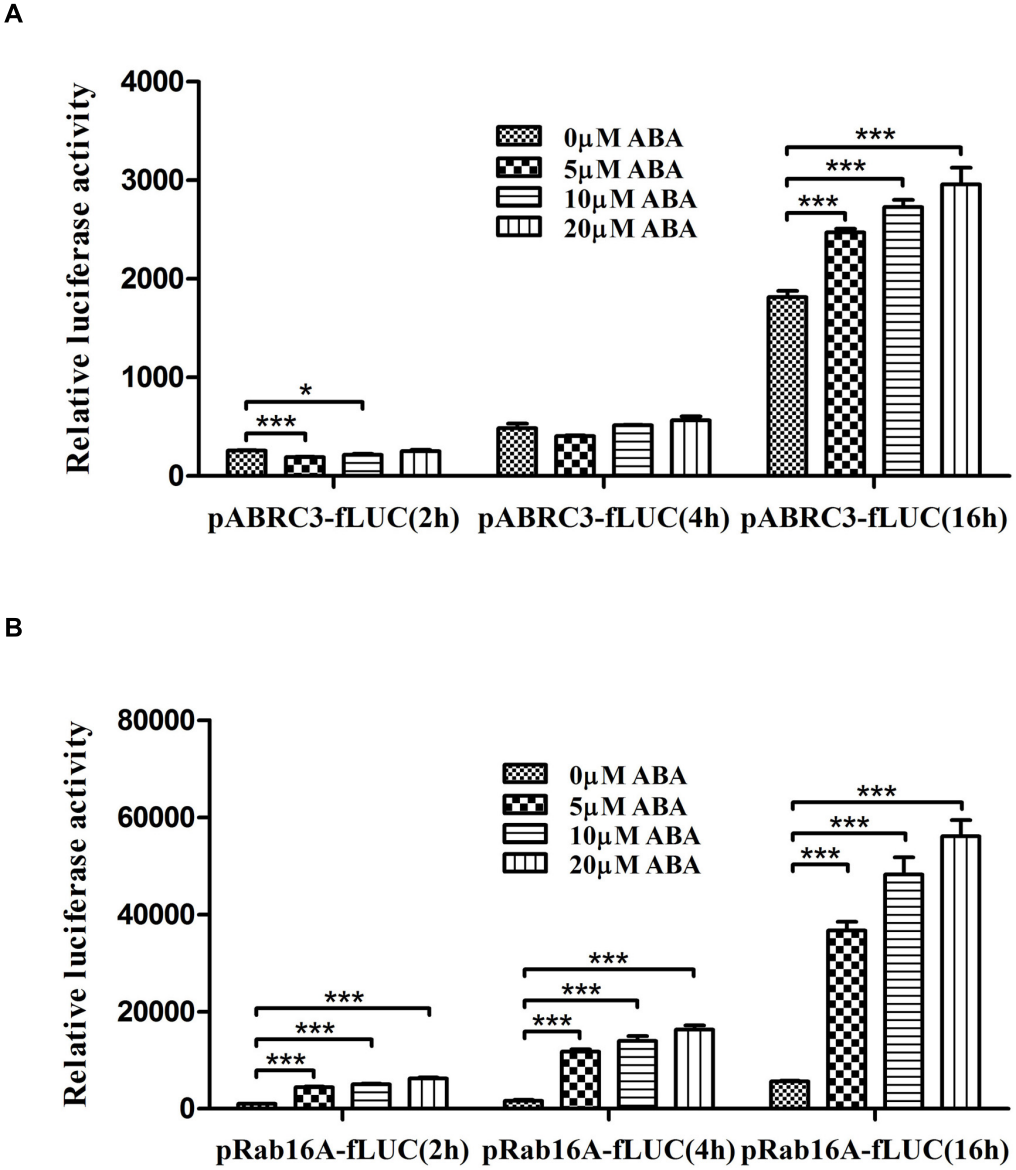
***Rab16A* promoter responds rapidly and significantly to ABA in rice protoplasts.** Dual luciferase assay driven by ABA-responsive promoters. **(A)** pABRC3-fLUC reporter. **(B)** pRab16A-fLUC reporter. The mean value of relative luciferase activity for three independent experiments is shown, and error bars indicate SD; analysis of variance (ANOVA) with Tukey’s test, ^∗^*P* < 0.05, ^∗∗∗^*P* < 0.001.

The first step to investigate ABA-dependent gene expression regulation using TGERP is to establish ABA-responsive reporter systems. Accordingly, we constructed a reporter vector consisting of *Rab16A* promoter fused with fLUC. The reason for using *Rab16A* promoter is that it has been known as a representative ABA-responsive marker gene in rice (Mundy and Chua, 1988; Mundy et al., 1990; Nakagawa et al., 1996; Miyoshi et al., 1999; Xu et al., 2006; Lu et al., 2009; Kim et al., 2012; Joo et al., 2014). We examined the ABA-responsive induction of fLUC using pRab16A-fLUC and pABRC3-fLUC, which has been used as a control compared to pRab16A. Both pABRC3-fLUC and pRab16A-fLUC were individually transfected with pAtUBQ-rLUC as internal control and transiently over-expressed for 2, 4, and 16 h in the presence of 0, 5, 10, and 20 μM ABA in rice protoplasts. As shown in **Figure 1A**, pABRC3-fLUC expression was not induced under any concentration of ABA at 2 and 4 h. However, as ABA concentration increased from 5 to 20 μM, pABRC3-fLUC expression at 16 h was induced 36, 50, and 63%, respectively. By contrast, pRab16A-fLUC expression was induced under ABA treatments beginning at 2 h (**Figure 1B**). Thus, ABA treatments led to rapid and significant induction of pRab16A-fLUC expression compared to the pABRC3-fLUC expression under the same conditions. In particular, the increasing rate of pRab16A-fLUC induction by addition of 5 μM ABA, that is 4.4-, 7.2- and 6.5-fold at 2, 4, and 16 h, respectively, was more efficient compared to that of pRab16A-fLUC induction by addition of 10 and 20 μM ABA suggesting that 5 μM ABA was sufficient to induce pRab16A-fLUC expression (**Figure 1B**). When we compared the increase of fLUC expression at each time under different ABA concentrations, the relative rate of fLUC induction was very similar at 4 and 16 h. Induction rates were 7.2-, 8.6-, and 10-fold at 4 h, and 6.5-, 8.6-, and 10-fold at 16 h under 5, 10, 20 μM ABA conditions, respectively (**Figure 1B**). However, at 2 h, fLUC was induced 4.4-, 5-, and 6.9-fold (**Figure 1B**). These data suggest that the proper time to monitor the ABA-mediated regulation of gene expression using pRab16A-fLUC is 4 h after ABA treatment in TGERP. Overall, these results indicate that *Rab16A* promoter fused to fLUC can be used as a reporter system for ABA-dependent gene expression due to rapid and significant response to ABA in TGERP.

### Group A OsbZIPs Differentially Induce the Rab16A Promoter in TGERP Depending on ABA Concentration

**FIGURE 2:**
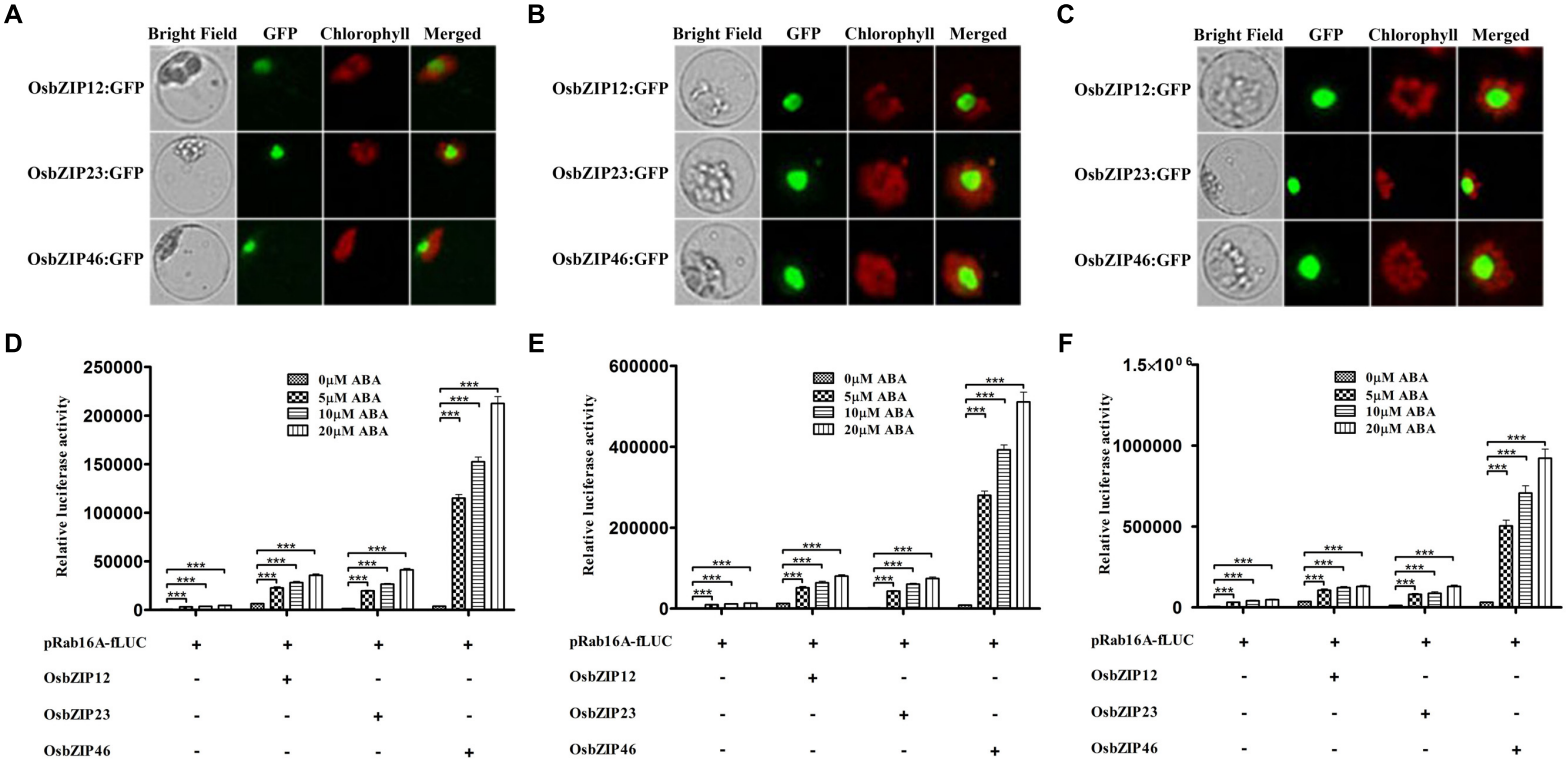
**OsbZIPs have differential *trans-*activation activity for *Rab16A* promoter in rice protoplasts. (A)** Expression analysis of OsbZIP12:GFP, OsbZIP23:GFP, and OsbZIP46:GFP in rice protoplasts. After transfection, protoplasts were incubated for **(A)** 2 h, **(B)** 4 h, and **(C)** 16 h. GFP signals of OsbZIP12:GFP, OsbZIP23:GFP, and OsbZIP46:GFP were detected after 2 h incubation and gradually increased. OsbZIP:GFPs were used at 10 μg per transfection. Exposure time of GFP fluorescence was 200 ms. Chlorophyll auto-fluorescence is in red to distinguish it from GFP (green) fluorescence. **(D–F)** Dual luciferase assay after 2 h **(D)**, 4 h **(E)**, and 16 h **(F)** incubations. His-tagged OsbZIP12, OsbZIP23, and OsbZIP46 were transfected with pRab16A-fLUC reporter plasmid and pAtUBQ-rLUC plasmid as an internal control into rice protoplasts by PEG transfection. After transfection, protoplasts were incubated for 2, 4, and 16 h in the presence of 0, 5, 10, and 20 μM ABA under light. The mean value of relative luciferase activity for three independent experiments is shown, and error bars indicate SD; ANOVA with Tukey’s test, ^∗∗∗^*P* < 0.001.

Group A OsbZIPs are major transcription factors regulating ABA-dependent gene expression (Lu et al., 2009; Amir Hossain et al., 2010; Yang et al., 2011; Joo et al., 2014). In case of TRAB1 (OsbZIP66), it induced more expression of luciferase reporter gene through ABRC of *Osem* promoter in the presence of ABA using rice cultured-cell protoplasts (Hobo et al., 1999). Accordingly, we examined whether OsbZIP12, -23, and -46, already functionally characterized could induce the pRab16A-fLUC reporter in an ABA-dependent manner in TGERP as in whole-plant systems (Xiang et al., 2008; Amir Hossain et al., 2010; Yang et al., 2011; Tang et al., 2012; Joo et al., 2014; Park et al., 2015). First, we monitored how rapid OsbZIPs could be synthesized in TGERP. Reporter constructs representing genes from three different subclasses of group A OsbZIPs, namely OsbZIP12:GFP, OsbZIP23:GFP, and OsbZIP46:GFP, were transfected into protoplasts, and GFP fluorescence was observed at 2, 4, and 16 h. GFP fluorescence started to appear in the nucleus from 2 h and fluorescence intensity was strongly enhanced after 2 h until 16 h (**Figures 2A–C**). At 4 h, OsbZIP protein synthesis seemed to be at an exponential stage and it appeared that this was sufficient time to allow expression of protein in TGERP (**Figure 2B**).

When we examined the effects of OsbZIPs in terms of pRab16A-fLUC expression, OsbZIP12, -23, and -46 all enhanced the activities of the *Rab16A* promoter, both time and ABA-concentration dependently as shown in **Figures 2D–F**. However, the *trans*-activation activity among the three OsbZIPs was quite different. Representatively at 4 h OsbZIP12 induced the luciferase 5.4-, 5.7-, and 6.1-fold, OsbZIP23 induced luciferase 4.5-, 5.4-, and 5.6-fold and OsbZIP46 induced 29-, 34.8-, and 38.7-fold in 5, 10, and 20 μM ABA concentration, respectively (**Figure 2E**). These results indicate that OsbZIP46 has the strongest *trans*-activity in response to ABA among the three OsbZIPs for the *Rab16A* promoter. In conclusion, 5 μM ABA concentration and 4 h ABA treatment seems to be appropriate conditions to monitor ABA-dependent gene expression using pRab16A-fLUC reporter and OsbZIPs in TGERP.

### Over-Expression of SAPK2 can Increase *Trans*-Activity of OsbZIP46 Independent of ABA in Rice Protoplasts

**FIGURE 3:**
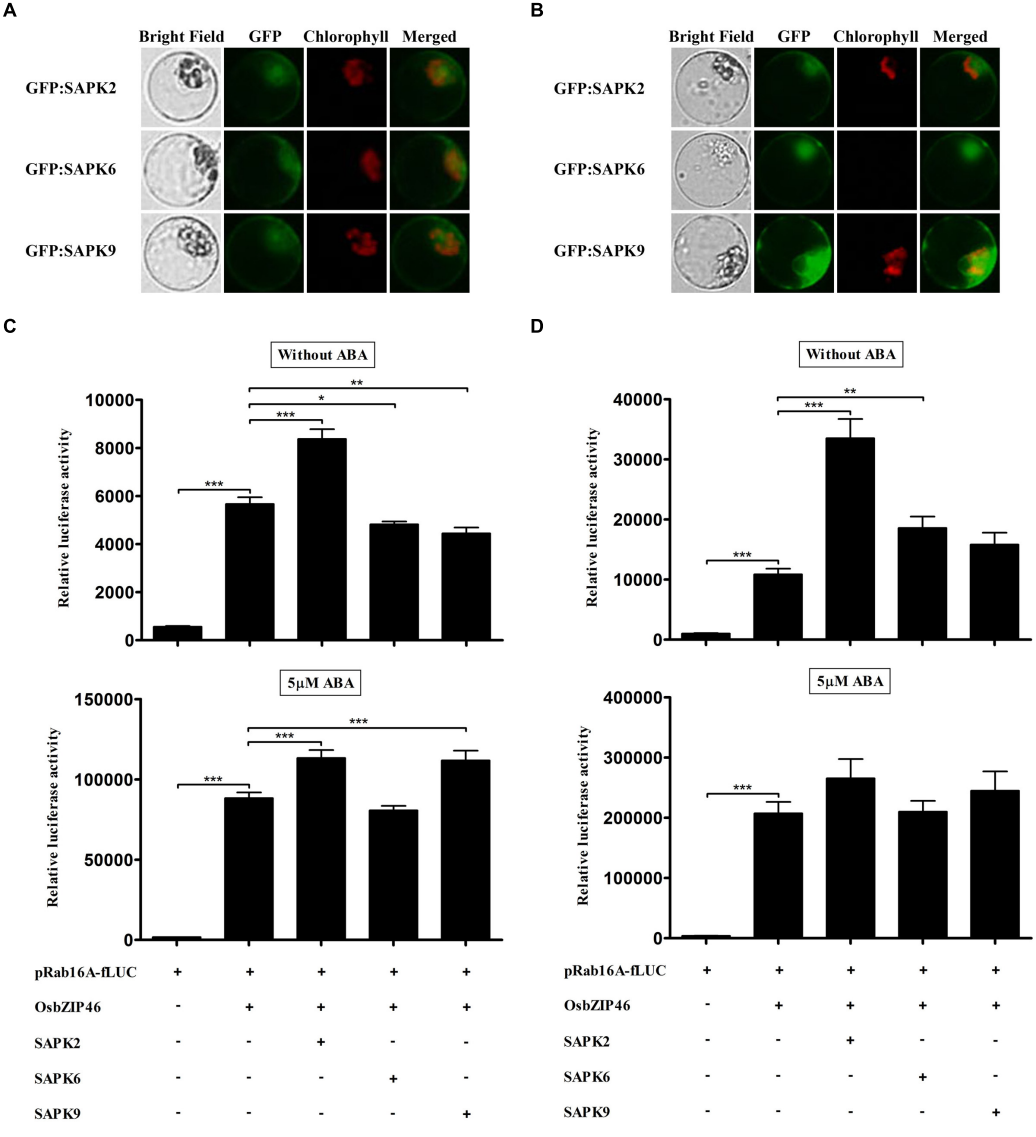
**Over-expression of SAPKs cannot increase *trans-*activation activity of OsbZIP46 significantly in wild-type rice protoplasts in the presence of ABA. (A,B)** Expression analysis of GFP:SAPK2, GFP:SAPK6, and GFP:SAPK9 in rice protoplasts. After transfection, protoplasts were incubated for **(A)** 2 h and **(B)** 4 h. GFP signal of GFP:SAPK2, GFP:SAPK6, and GFP:SAPK9 was detected after 2 h incubation and gradually increased. GFP:SAPKs were used at 10 μg per transfection. Exposure time of GFP fluorescence was 600 ms. Chlorophyll autofluorescence is in red to distinguish it from GFP (green) fluorescence. **(C,D)** Dual luciferase assay after 2 h **(C)** and 4 h **(D)** incubations. Flag-tagged SAPK2, -6, and -9 were transfected with HA-tagged OsbZIP46, pRab16A-fLUC reporter plasmid and pArUBQ-rLUC plasmid as an internal control. After transfection, protoplasts were incubated for 2 and 4 h in the presence of 0 and 5 μM ABA under light. The mean value of relative luciferase activity for three independent experiments is shown, and error bars indicate SD; ANOVA with Tukey’s test, ^∗^*P* < 0.05, ^∗∗^*P* < 0.01, ^∗∗∗^*P* < 0.001.

SAPKs can be classified into three different subclasses in terms of ABA-dependent kinase activity (Kobayashi et al., 2004; Kulik et al., 2011). SAPK2, -6, and -9 belonging to each subclass (I, II, and III, respectively) has been shown to bind OsbZIP46 directly and SAPK2 and -6 can phosphorylate OsbZIP46 without ABA in *in vitro* phosphorylation assay (Tang et al., 2012). However, transcriptional activity of OsbZIP46 enhanced directly by these SAPKs has not been confirmed yet. Thus we examined whether SAPK2, -6, and -9 could activate the OsbZIP46 in TGERP. Firstly, we confirmed whether the protein synthesis of SAPK2, -6, and -9 is enough at 2 and 4 h. GFP:SAPK2, GFP:SAPK6, and GFP:SAPK9 started to show much weaker GFP fluorescence after 2 h incubation than OsbZIP (**Figure 3A**) and GFP signal was significantly enhanced at 4 h for all SAPKs (**Figure 3B**). It seems that the expression of SAPKs required more induction time as compared to OsbZIPs. To examine the effects of SAPKs through the OsbZIP46 in the presence or absence of ABA, SAPK2, -6, and -9 were co-transfected with OsbZIP46, respectively. After 2 h incubations, fLUC expression was up to 48% greater with over-expression of SAPK2, whereas the over-expression of SAPK6 and -9 decreased fLUC expression without ABA (**Figure 3C**). After 4 h incubations, over-expression of SAPK2, -6, and -9 enhanced the fLUC expression by 3.1-, 1.7-, and 1.5-fold without ABA, respectively (**Figure 3D**). With 5 μM ABA at 2 h, over-expression of SAPK2 and -9 increased fLUC expression about 1.3-fold (**Figure 3C**). With 5 μM ABA at 4 h, over-expression of SAPK2, -6, and -9 increase fLUC expression about 1.3-, 1-, and 1.2-fold, respectively, but one-way ANOVA showed no significant effects (**Figure 3D**). Taken together, in the absence of ABA, SAPK2 can activate OsbZIP46 most significantly among three different subfamilies of SAPKs.

### OsPP2C6 and -8 can Suppress the *Trans*-Activity of OsbZIP46 Completely through Inactivation of SAPK9 but Show Differential Characteristics in TGERP

**FIGURE 4:**
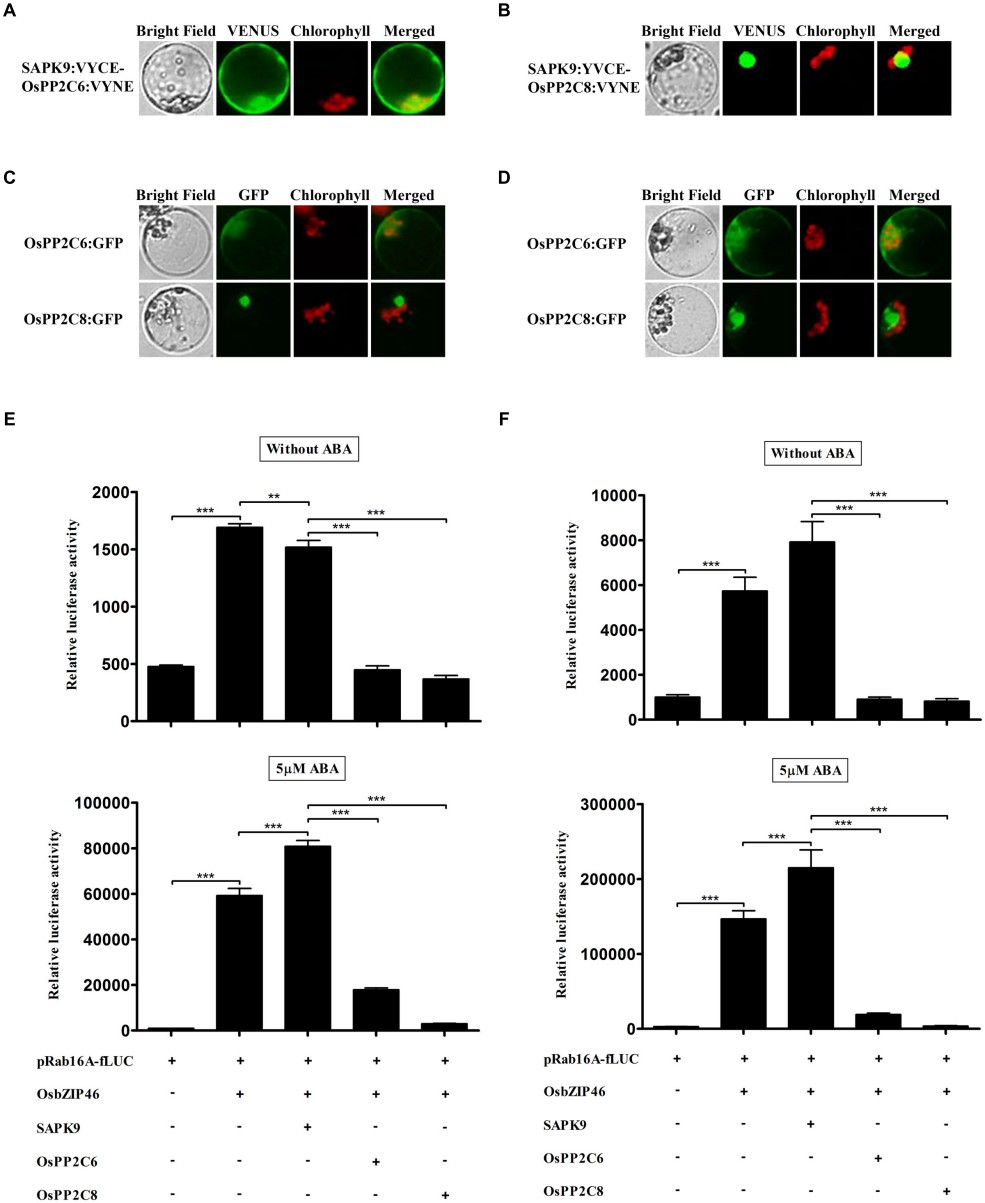
**OsPP2Cs completely suppress the *trans*-activity of OsbZIP46 in rice protoplasts. (A,B)** Interactions of SAPK9 with OsPP2C6 and OsPP2C8 in rice protoplasts. The interaction of SAPK9 with OsPP2C6 and OsPP2C8 was detected by BiFC analysis. SAPK9 interacts with OsPP2C6 in both the nucleus and cytosol and with OsPP2C8 in the nucleus. **(C,D)** Expression analysis of OsPP2C6:GFP and OsPP2C8:GFP in rice protoplasts. After transfection, protoplasts were incubated for **(C)** 2 h and **(D)** 4 h. GFP signal of OsPP2C6:GFP and OsPP2C8:GFP was detected after 2 h incubation and gradually increased. GFP:OsPP2Cs were used at 10 μg per transfection. Exposure time of GFP fluorescence was 400 ms. Chlorophyll autofluorescence is in red to distinguish it from GFP (green) fluorescence. **(E,F)** Dual luciferase assay after 2 h **(E)** and 4 h **(F)** incubations. HA-tagged OsPP2C6 and -8 were transfected with HA-tagged OsbZIP46, Flag-tagged SAPK9, pRab16A-fLUC reporter plasmid and pAtUBQ-rLUC plasmid as an internal control. After transfection, protoplasts were incubated for 2 and 4 h in the presence of 0 and 5 μM ABA under light. The mean value of relative luciferase activity for three independent experiments is shown, and error bars indicate SD; ANOVA with Tukey’s test, ^∗∗^*P* < 0.01, ^∗∗∗^*P* < 0.001.

Some SnRK2s, *Arabidopsis* orthologs of rice SAPKs, have previously been shown to bind directly to several group A PP2Cs and are inactivated by PP2C-mediated dephosphorylation in *Arabidopsis* (Umezawa et al., 2009; Vlad et al., 2009; Soon et al., 2012; Xie et al., 2012). Therefore, we examined whether SAPK9 interacts with subclass A PP2Cs of rice using BiFC experiments in rice protoplasts (**Figures 4A,B**). Interestingly, SAPK9 interacted with two OsPP2Cs showing different subcellular localizations of complexes; the complex between OsPP2C8 and SAPK9 was localized in nucleus and the complex of OsPP2C6 and SAPK9 was observed in cytosol and nucleus, depending on the subcellular localization of the OsPP2Cs (**Figures 4A–D**). Yeast two hybrid assay also showed SAPK9 interacted with OsPP2C6 and OsPP2C8 (data not shown). The OsPP2Cs proteins showed much stronger expression than SAPKs in terms of GFP fluorescence (**Figures 4C,D**). We also monitored fLUC expression in dual luciferase assays to characterize the effects of OsPP2Cs on ABA-dependent gene expression. After 2 h incubation, over-expressed OsPP2C6 and OsPP2C8 decreased the fLUC expression to about 70 and 76%, as compared to the fLUC expression without OsPP2C in the absence of ABA. In the 5 μM ABA condition, the expression was decreased to about 78 and 96%, respectively (**Figure 4E**). At 4 h, the effects on luciferase activity of OsPP2C6 and OsPP2C8 were similar to those at 2 h, but inhibition activity was stronger. OsPP2C6 and OsPP2C8 decreased the fLUC expression to about 89 and 90% in the absence of ABA and about 91 and 98% in the presence of 5 μM ABA at 4 h (**Figure 4F**). Overall, our results showed that OsPP2C6 and -8 have similar inhibition activity of the fLUC expression in absence of ABA, but OsPP2C8 has stronger inhibition activity than OsPP2C6 in the presence of ABA.

### OsPYL/RCARs have Differential Activities for ABA-Dependent Suppression of OsPP2Cs in TGERP

**FIGURE 5:**
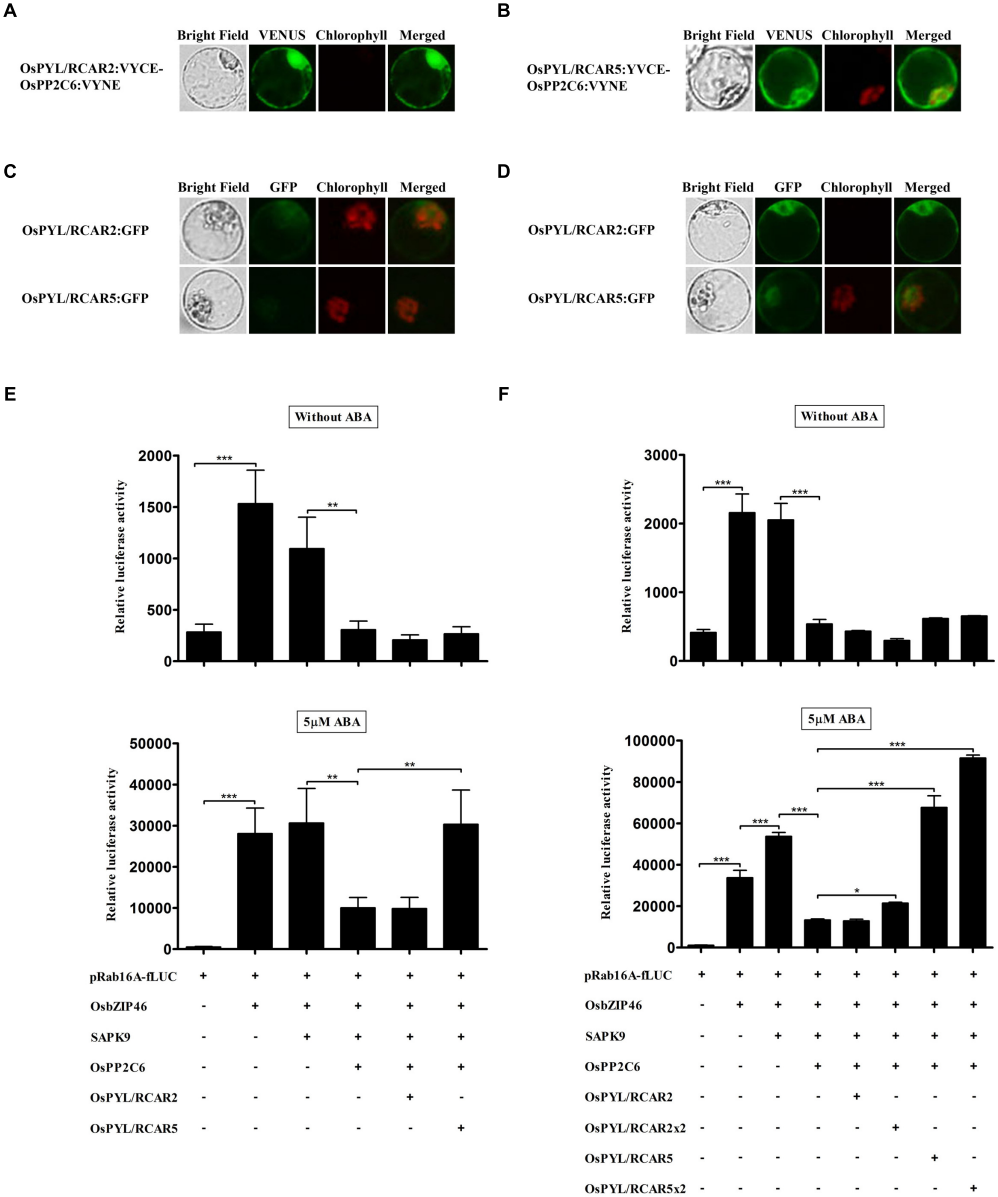
**OsPYL/RCARs differentially increase ABA-dependent signaling outputs in rice protoplasts. (A,B)** Interactions of OsPP2C6 with OsPYL/RCAR2 and OsPYL/RCAR5 in rice protoplasts. The interaction of OsPP2C6 with OsPYL/RCAR2 and OsPYL/RCAR5 was detected by BiFC analysis. OsPP2C6 interacts with OsPYL/RCAR2 and OsPYL/RCAR5 in both the nucleus and cytosol. **(C,D)** Expression analysis of OsPYL/RCAR2:GFP and OsPYL/RCAR5:GFP in rice protoplasts. After transfection, protoplasts were incubated for **(C)** 2 h and **(D)** 4 h. GFP signal of OsPYL/RCAR2:GFP and OsPYL/RCAR5:GFP was detected very weakly after 2 h incubation and gradually increased. OsPYL/RCAR:GFP were used at 10 μg per transfection. Exposure time of GFP fluorescence was 600 ms. Chlorophyll autofluorescence is in red to distinguish it from GFP (green) fluorescence. **(E,F)** Dual luciferase assay after 2 h **(E)** and 4 h **(F)** incubations. Flag-tagged OsPYL/RCAR2 and -5 were transfected with HA-tagged OsbZIP46, Flag-tagged SAPK9, HA-tagged OsPP2C6, pRab16A-fLUC reporter plasmid and pAtUBQ-rLUC plasmid as an internal control. After transfection, protoplasts were incubated for 2 and 4 h in the presence of 0 and 5 μM ABA under light. The mean value of relative luciferase activity for three independent experiments is shown, and error bars indicate SD; ANOVA with Tukey’s test, ^∗^*P* < 0.05, ^∗∗^*P* < 0.01, ^∗∗∗^*P* < 0.001.

In ABA-dependent gene expression signaling, ABA receptors, PYL/RCARs interact with subclass A PP2Cs and inhibit its activity in *Arabidopsis* and rice (Park et al., 2009; Hao et al., 2011; Mosqunaa et al., 2011; Antoni et al., 2012; Kim et al., 2012; Zhao et al., 2013; He et al., 2014). These PYL/RCARs can be classified into dimer and monomer receptors, which have different characteristics (Hao et al., 2011; Okamoto et al., 2013; He et al., 2014). We found that both OsPYL/RCAR2 (the dimer form ABA receptor) and OsPYL/RCAR5 (the monomer form) could interact with OsPP2C6 in BiFC analysis (**Figures 5A,B**). Both ABA receptors were expressed in cytosol and nucleus, and the proteins were expressed enough to monitor at 4 h (**Figures 5C,D**). To examine the ABA receptor activity, we co-transfected all of the ABA signaling components from OsbZIP transcription factor to ABA receptor and reconstituted ABA signaling in rice protoplasts. As shown in **Figures 5E,F**, over-expression of OsPYL/RCAR2 and OsPYL/RCAR5 did not significantly change fLUC expression in the absence of ABA, at 2 and 4 h. However, in the presence of 5 μM ABA, over-expression of OsPYL/RCAR5 led to significant induction of fLUC, which increased threefold at 2 h and fivefold at 4 h. By contrast, OsPYL/RCAR2 did not induce fLUC at 2 or 4 h in the presence of 5 μM ABA (**Figures 5E,F**). In addition, when more DNA of OsPYL/RCAR2 and -5 was transfected, fLUC expression was increased in the presence of ABA. These results show that OsPYL/RCAR2 and OsPYL/RCAR5 have similar interaction partners but have different signaling effects dependent on ABA concentration.

## Discussion

After the complete sequencing of the rice genome, re-sequencing of rice cultivars and genomic resources have given blue print of rice genome for researchers and breeders (International Rice Genome Sequencing Project, 2005; Xu et al., 2012). Also bioinformatics analysis tools and database development to compare the genomes, transcriptomes, proteomes and metabolomes have opened the era of systems biology (Sato et al., 2011). However, functionally identified genes remain relatively few and molecular mechanisms of signaling pathways identified systematically are also lacking in rice. The slow progress in functional genomics of rice compared to other -omics such as structural genomics, proteomics, and metabolomics are related to the time-consuming and laborious genetic analysis methods for the whole plant. Thus, transient functional identification systems are required for high-throughput analysis in rice and other crops for functional and systematic research in the new functional genomics era.

Several research groups have reported successful rice protoplast isolation from stem and sheath of young green seedlings, different from the green leaves used in *Arabidopsis* and maize (Chen et al., 2006, 2015; Zhang et al., 2011). These differences are related to different leaf anatomy; rice has very thin leaves with very few mesophyll cells. Protoplasts have been used as protein expression systems in various species. In tobacco and soybean, for instance, CAT activity was detected 30 min and 6 h after transfection, respectively (Grosset et al., 1990). In our TGERP, all proteins were detectable by 2 h after transfection based on GFP fluorescence, although the expression intensity was quite different among proteins. The proteins accumulated proportionally to incubation times from 2 to 16 h. Thus, signaling effects of genes could be monitored between 4 and 16 h after transfection in our TGERP.

In *Arabidopsis*, there are several different reporter systems that consist of promoter or *cis*-elements responsive to the signal of interest and reporter genes such as LUC, GUS, and GFP (Sheen, 2001; Yoo et al., 2007). The *RD29B* promoter was used as a reporter for reconstitution of ABA signaling in *Arabidopsis* (Fujii et al., 2009). The *RD29B* promoter contains the ABRE and is known to respond specifically to ABA in *Arabidopsis* mesophyll protoplasts (Nakashima et al., 2006; Msanne et al., 2011). However, when we used pABRC3-fLUC reporter system, which contains a synthetic promoter (ABRC3) consisting of an ABRE, Coupling element 3 (CE3) and 35S minimal promoter (**Supplementary Figure S1**), it was not very responsive to ABA in our TGERP. By contrast, pRab16A-fLUC reporter system showed rapid and strong responsiveness to ABA by itself as well as by OsbZIP like RD29B promoter in *Arabidopsis*. This result suggests that *cis*-elements other than ABRE might play roles in ABA responsiveness in the *Rab16A* promoter.

In *Arabidopsis* protoplasts, SnRK2.6 activates ABF2 (abscisic acid response elements binding factor 2) and induces the expression of luciferase fused with *RD29B* promoter more than fivefold under ABA treatment (Fujii et al., 2009). However, OsbZIP46 was not significantly activated by SAPKs under ABA treatment conditions in our TGERP. This finding suggests that there might be sufficient endogenous SAPKs to activate over-expressed OsbZIPs fully in wild-type rice protoplasts treated with ABA. Kobayashi et al. (2004) reported that SAPK2 is rapidly activated by osmotic stress whereas SAPK9 is tightly regulated by ABA. In our TGERP, SAPK2 could activate OsbZIP46 in the absence of ABA. In contrast to SAPK2, SAPK9 could not activate OsbZIP46 in the absence of ABA. These findings suggest that SAPK2 might be activated by osmotic stress in rice protoplasts and that SAPK9 might be more tightly regulated by ABA compared to SAPK2. Thus, our TGERP showed signaling effects of SAPKs similar to those in plants in the absence of ABA.

He et al. (2014) classified rice ABA receptors into monomer and dimer forms. It was previously reported that dimer-form receptors could not suppress the activity of OsPP2Cs ABA-independently *in vitro* but monomer-form receptors could suppress the activity of OsPP2Cs ABA-independently according to the concentrations of OsPYLs and OsPP2Cs *in vitro*. In our experiments BiFC results showed ABA independent interaction between OsPYL/RCAR2 and 2 with OsPP2C2 respectively. And we also confirmed that OsPP2C6 interacted with OsPYL/RCAR2 in the absence of ABA (data not shown). However, we found that dimer-form OsPYL/RCAR2 and monomer-form OsPYL/RCAR5 both failed to suppress OsPP2C6 in the absence of ABA. Even in the presence of ABA, the dimer-form OsPYL/RCAR2 was required in higher concentrations than OsPYL/RCAR5 to suppress OsPP2C6 activity and moreover the suppressor activity of OsPYL/RCAR2 was quite low as compared to OsPYL/RCAR5 that completely suppressed OsPP2C6 activity in the presence of 5 μM ABA. Thus, we can monitor the different ABA sensitivities among receptors *in vivo* experiments in terms of suppression of OsPP2C by OsPYL/RCARs. Such kinds of differences imply that OsPYL/RCARs might participate differentially in ABA signaling pathway depending on the cellular ABA concentrations. In summary, we successfully developed a monitoring system for ABA signaling in rice protoplasts and reconstituted the signaling components to demonstrate similar signaling characteristics as previously reported for whole plants. Thus, we showed that transient gene expression systems in rice protoplasts are suitable not only for functional analysis of single genes but also for characterization of signaling pathways or gene networks. This system therefore represents very useful technology to study functional genomics in rice or other monocots.

## Author Contributions

NK, S-JM, and B-GK designed the research. NK and E-HC cloned constructs. NK carried out dual luciferase assay. S-JM and EK performed the BiFC and GFP analysis. MM and J-AK setup the rice protoplast PEG transformation methods. IY, M-OB, S-DY revised the manuscript. NK, S-JM, and B-GK analyzed the data and wrote the manuscript. All authors read and approved the manuscript.

## Conflict of Interest Statement

The authors declare that the research was conducted in the absence of any commercial or financial relationships that could be construed as a potential conflict of interest.

## Supplementary Material

Figure S1 **Schematic diagram of constructs used in this study. (A,B)** ABA-responsive reporter vectors used in TGERP. Nucleotide sequences of the *HVA1* promoter fragment of barley **(A)** and full length *Rab16A* promoter of rice **(B)** containing ACGT (red) and non-ACGT (blue) core sequences. ABA-responsive elements are underlined. **(C)** Internal vector used in the transient assays.
