# Supplementary figures and images for: Functional characterization and reconstitution of ABA signaling components using transient gene expression in rice protoplasts
Source: Front Plant Sci. 2015 Aug 5;6:614. doi: 10.3389/fpls.2015.00614 (PMC4524894; doi:10.3389/fpls.2015.00614)

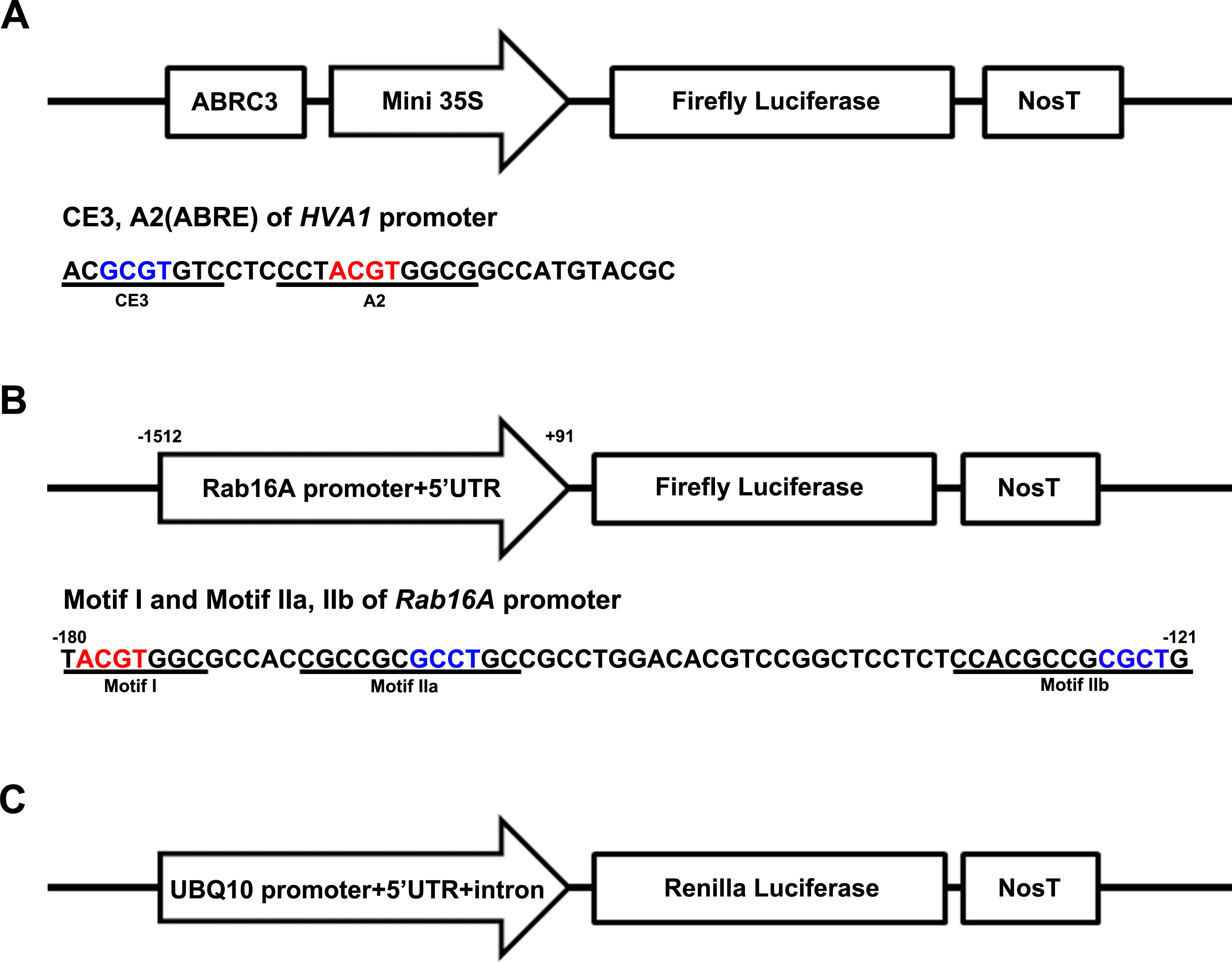

Supplement: Figure S1 — Schematic diagram of constructs used in this study. (A,B) ABA-responsive reporter vectors used in TGERP. Nucleotide sequences of the HVA1 promoter fragment of barley (A) and full length Rab16A promoter of rice (B) containing ACGT (red) and non-ACGT (blue) core sequences. ABA-responsive elements are underlined. (C) Internal vector used in the transient assays. [file Image_1.JPEG]
